# Trends, challenges and ethical considerations in pediatric robotic surgery

**DOI:** 10.1016/j.sopen.2025.12.002

**Published:** 2025-12-18

**Authors:** Anoush Sardesai Sadat, Suhaib J.S. Ahmad, Sjaak Pouwels

**Affiliations:** a Kasturba Medical College, Manipal, Manipal Academy of Higher Education, Manipal, 576104, Karnataka, India; b Department of General Surgery, Betsi Cadwaladr University Health Board, Wales, United Kingdom; c Department of Emergency Medicine, Inselspital University of Bern, Bern, Switzerland; d Department of Surgery, University Hospital OWL of Bielefeld University – Campus Klinikum Lippe, Detmold, NRW, Germany; e Department of Intensive Care Medicine, Elisabeth-Tweesteden Hospital Tilburg, the Netherlands

**Keywords:** Pediatric surgery, Ethics, Robotic surgery

## Abstract

Robotic surgery is revolutionizing healthcare by offering unparalleled precision and control in minimally invasive procedures. With the da Vinci system leading this transformation, surgeons can perform complex operations with enhanced accuracy, reduced recovery times, and fewer complications. In this narrative review, we expanding role of robotic surgery in pediatric cases, highlighting its advantages over conventional techniques, such as improved visualization, reduced tremor, and shorter learning curves. However, challenges like high costs, limited instrument availability, and ethical concerns about access and equity persist. We also examine emerging trends, including telesurgery and augmented reality, which promise to further innovate the field. As pediatric robotic surgery continues to evolve, balancing technological advancements with ethical considerations is crucial to ensuring all children benefit from these cutting-edge surgical solutions. Understanding these dynamics will help guide future applications, making robotic surgery not just a tool for select cases but a standard of care that is accessible, efficient, and equitable.

## Introduction

Minimally Invasive Surgery (MIS) has become one of the most significant advancements in modern medicine, transforming the way many procedures are performed. Among the various MIS techniques—such as laparoscopy, endoscopy, and arthroscopy—Robotic Surgery has emerged as a particularly groundbreaking innovation. This approach has elevated the precision, control, and outcomes of surgical procedures, establishing a new standard in the field. Central to this transformation is the da Vinci robotic system (Intuitive Surgical, Sunnyvale, CA, USA), an FDA-approved system for robotic surgery. Its application has led to significant progress across a range of procedures, including cholecystectomy, hernia repair, fundoplication, bariatric surgeries, and oncological operations [1,2].

The introduction of robotic systems has not only enhanced the technical aspects of surgery but also paved the way for better patient outcomes, including reduced recovery times and fewer complications. In this article, we explore the application of robotic surgery within pediatric surgical care, examining both its potential benefits and the ethical considerations that arise when using such technology to treat young patients.

## Robotic surgery in children

Pediatric surgical conditions encompass a broad spectrum of health challenges, including infections, injuries, cancers, and congenital abnormalities. These conditions highlight the complexity and diversity of issues that can arise in children, necessitating specialized surgical interventions. Pediatric surgery is essential in managing these varied challenges. Each condition presents unique demands, requiring a deep understanding of the distinct physiological characteristics of children to ensure that surgical care is effective and tailored to their specific needs [3].

Robotic surgery offers several significant advantages in pediatric procedures compared to conventional laparoscopic surgery. One of the primary benefits is the high-resolution, 3-dimensional vision it provides, offering surgeons a clearer and more detailed view of the surgical field which is an essential feature for delicate pediatric operations. The system's intuitive movement with tremor control allows for more precise maneuvers, reducing the risk of errors caused by natural hand tremors. Additionally, robotic instruments provide seven degrees of freedom, closely mimicking the full range of motion of the human hand, which enables surgeons to perform complex tasks with greater ease. Motion scaling further enhances precision by allowing for smaller, more controlled movements. Improved ergonomic features also reduce physical strain on surgeons during lengthy procedures, leading to better performance and less fatigue. Moreover, the learning curve for mastering robotic surgery is shorter compared to traditional laparoscopic techniques, making it more accessible for surgeons to adopt and apply these advanced methods in pediatric care [[4], [5], [6]].

**Fig. 1:**
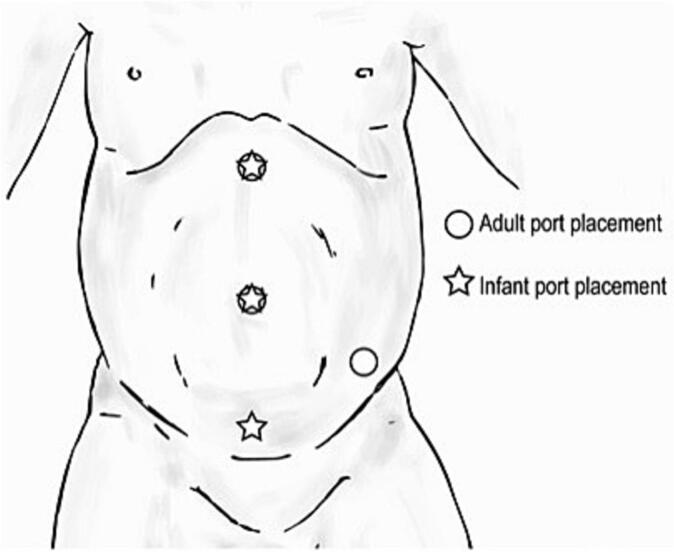
Trocar positioning for upper urinary tract surgery in infants (✩) compared to that for adults (○) [8].

However, despite these advantages, robotic surgery in pediatric procedures comes with certain limitations. The large size of the robotic system can be challenging to accommodate in the smaller operating rooms typically used for pediatric surgeries (Fig. 1). Additionally, the limited selection of instruments specifically designed for pediatric use can restrict the range of procedures that can be performed robotically. Lengthy setup and docking times further add to the overall duration of the surgery, which is particularly concerning in pediatric cases where minimizing anesthesia time is crucial [7]. The use of 5 mm instruments, while necessary for smaller patients, offers a more restricted range of motion compared to larger instruments, potentially impacting surgical precision. Moreover, the high capital investment required to purchase the robotic system, along with ongoing maintenance costs and the expense of disposable items, can pose a significant financial burden on healthcare facilities, making robotic surgery less accessible for some institutions and patients [[4], [5], [6], [7]].

There are relevant risks associated with robotic surgery, including potential computer system failures that could interrupt the surgical procedure, and the possibility of losing communication between the surgeon and the operating room, which are risks that are heightened in telesurgery and remote robotic surgery. There is also the risk of encountering intraoperative complications that require urgent attention. Furthermore, data transmission during robotic procedures might face delays or losses, especially over long distances or when multiple network types, such as integrated services digital networks or asynchronous transfer mode networks, are involved. These concerns highlight the importance of having reliable systems and contingency plans in place to mitigate these risks and ensure the smooth execution of robotic surgeries [4].

At present, robotic systems are extensively utilized in pediatric surgeries to carry out a wide range of procedures that were traditionally managed through laparoscopic and thoracoscopic techniques. These advanced robots enable surgeons to employ minimally invasive approaches across various operations, offering enhanced precision and control when treating young patients. In the early 2000s, robotic surgeries such as fundoplication and pyeloplasty were initially performed in Europe and the USA, marking the beginning of this technological shift in pediatric surgery [7,9].

**Fig. 2:**
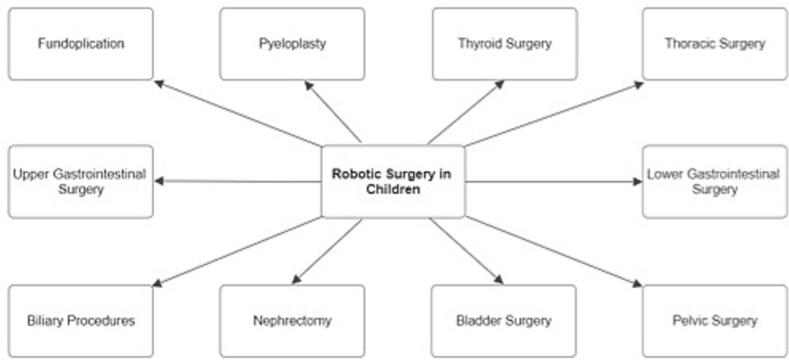
Indications of robotic surgery in pediatric patients [7,9,10].

Recent studies further highlight the expanding role of pediatric robotic surgery, demonstrating its application in an increasingly diverse array of procedures (Fig. 2). These include performing fundoplications to treat gastroesophageal reflux, pyeloplasty for correcting ureteropelvic junction obstructions, and intricate thyroid surgeries. Additionally, robotic systems are now being utilized in thoracic surgeries, managing both upper and lower gastrointestinal conditions, and conducting complex biliary procedures like the removal of choledochal cysts and the treatment of biliary atresia. The versatility of robotic technology is also evident in its growing use in surgeries involving the kidneys, bladder, and pelvic region, underscoring its effectiveness in a broad spectrum of pediatric surgical interventions [7].

Research [[1], [2], [3], [4], [5], [6], [7], [8], [9], [10]] underscores the broad applicability and flexibility of robotic surgery within the realm of pediatric surgical care. It demonstrates how this advanced technology can be effectively utilized across a wide range of procedures, showcasing its potential to significantly improve surgical outcomes for young patients. By highlighting the various ways in which robotic systems can be adapted to meet the unique challenges of pediatric surgery, these studies emphasize the growing role of robotics in enhancing precision, reducing recovery times, and minimizing complications in pediatric operations. This versatility is a key factor in the increasing adoption of robotic surgery in the treatment of complex pediatric conditions, further solidifying its place as a transformative tool in modern surgical practice.

## Is the trend changing?

Robotic platforms offer significant advancements in pediatric surgery, thanks to their enhanced magnification, improved ergonomics, and increased precision. These systems provide surgeons with superior control and visibility compared to traditional methods, making it possible to perform more complex procedures with minimal invasiveness. While telesurgery—performing surgeries remotely—remains a futuristic concept due to substantial technical challenges, ongoing developments indicate its potential feasibility. Tele-mentoring programs, which enable experienced surgeons to guide less experienced ones from a distance, have already demonstrated the benefits of such technology. These programs have shown that remote assistance can enhance surgical education and expand access to expert knowledge, setting the stage for broader applications of robotic surgery in the future [1].

Additionally, research conducted in extreme environments like space missions and military operations could drive further advancements in robotics. The integration of image guidance and augmented reality into robotic-assisted surgeries represents a significant breakthrough, potentially improving safety and addressing challenges associated with minimally invasive techniques [1].

## Ethical aspects of pediatric robotic surgery

We believe that robotic surgery represents a promising advancement in delivering high-standard pediatric surgical care when appropriately indicated. The precision, control, and minimally invasive nature of robotic platforms can significantly enhance surgical outcomes and reduce recovery times. However, this technology introduces several ethical considerations that must be addressed. The high costs associated with robotic systems, along with the extended setup and operation times, may limit access and could potentially exacerbate existing disparities in healthcare.

From an ethical standpoint, it is crucial to ensure that the benefits of robotic surgery are not confined to a privileged few but are accessible to all patients in need. This means addressing the financial barriers that could restrict access and considering how to integrate these advanced technologies in a way that promotes fairness and equity. Striking a balance between leveraging cutting-edge technology and ensuring it is available to a broad patient base is essential to avoid reinforcing inequalities within the healthcare system. By addressing these ethical concerns, we can work towards a model of care that maximizes the advantages of robotic surgery while promoting inclusivity and justice in pediatric healthcare.

In our opinion, the higher costs associated with robotic surgery, especially where there is no significant reduction in hospital stay, highlight the need for a careful evaluation of its value in pediatric care. While robotic technology offers notable benefits, such as enhanced precision and potentially quicker recovery times, these advantages must be weighed against the financial implications. To justify the increased expenditure, it is crucial to assess whether the improved outcomes and patient satisfaction genuinely outweigh the additional costs. Transparent analysis and evidence-based assessments will be essential in determining if the benefits of robotic surgery in children merit its higher price tag.

## Conclusion

Robotic surgery represents a significant advancement in pediatric care, enhancing precision and outcomes for complex procedures. The da Vinci system has improved surgical techniques and patient recovery. However, its high costs and extended setup times present challenges, potentially limiting access and increasing healthcare disparities.

## CRediT authorship contribution statement

**Anoush Sardesai Sadat:** Writing – review & editing, Writing – original draft, Methodology, Investigation, Data curation, Conceptualization. **Suhaib J.S. Ahmad:** Writing – review & editing, Writing – original draft, Methodology, Formal analysis, Data curation, Conceptualization. **Sjaak Pouwels:** Writing – review & editing, Writing – original draft, Methodology, Investigation, Formal analysis, Data curation, Conceptualization.

## Consent to participate

Not applicable.

## Ethical approval statement

Not applicable.

## Funding sources

This study was not funded.

## Declaration of competing interest

The authors have no conflict of interest to declare.
